# Functional Outcomes of Pilon Fractures Treated by External Fixation, Delayed Plating, and Open Reduction and Internal Fixation (ORIF): A Prospective Cohort Study

**DOI:** 10.7759/cureus.74867

**Published:** 2024-11-30

**Authors:** Nayan Shrivastava, Muhammad Mannan, Muhammad A Hamid, Rizwan Akbar, Rudra M Prabhu

**Affiliations:** 1 Trauma and Orthopedic, Sancheti Institute for Orthopaedic and Rehabilitation, Pune, IND; 2 Trauma and Orthopaedics, University Hospitals Birmingham NHS Foundation Trust, Birmingham, GBR; 3 Trauma and Orthopaedics, Sheikh Zayed Medical College and Hospital, Rahim Yar Khan, PAK; 4 Orthopaedic Surgery, University Hospitals Birmingham NHS Foundation Trust, Birmingham, GBR; 5 Orthopaedic Surgery, Government Medical College, Srinagar, Srinagar, IND; 6 Trauma and Orthopaedics, Hayatabad Medical Complex Peshawar, Peshawar, PAK; 7 Orthopaedics, University Hospitals Birmingham NHS Foundation Trust, Birmingham, GBR; 8 Orthopaedics, King Edward Memorial Hospital and Seth Gordhandas Sunderdas Medical College, Mumbai, IND

**Keywords:** american orthopaedic foot and ankle society (aofas) score, external fixation, foot and ankle disability index (fadi), open reduction and internal fixation (orif), pilon fracture

## Abstract

Objective: This study aimed to evaluate the functional outcomes of three surgical management strategies for pilon fractures, including primary external fixation with delayed plating, external fixation with minimal internal fixation, and single-stage open reduction and internal fixation (ORIF) with plating.

Methods: This prospective cohort study included 34 patients with complex intra-articular fractures of the distal tibia (AO-OTA type 43-C) treated between June 2018 and December 2019. Patients were managed surgically based on the local skin condition and swelling, employing either primary-stage external fixation with delayed plating (Group A), external fixation with minimal internal fixation (Group B), or single-stage ORIF with plating (Group C). Patients were followed up at regular intervals (six weeks, three months, six months, and 12 months post-surgery). Functional outcomes were assessed using the American Orthopaedic Foot and Ankle Society Hindfoot Score (AOFAS) and Foot and Ankle Disability Index (FADI). Statistical significance was set at p<0.05.

Results: The mean age of patients was 41±12.34 years. Group C included 23 patients (67.7%), Group B included five patients (14.7%), and Group A included six patients (17.6%). There were no significant differences between groups regarding the mechanism of injury, hospital stay duration, complications, or final functional scores (p>0.05). Group C showed significantly better AOFAS and FADI scores at six weeks and three months compared to the other groups; however, by six and 12 months, no significant differences were observed between the groups.

Conclusion: While ORIF with plating (Group C) demonstrated superior short-term functional outcomes, no long-term differences were observed between the three surgical approaches for pilon fractures. These findings suggest that all three methods can be viable options, with choice depending on individual patient factors and surgeon preference.

## Introduction

Pilon fractures, characterized by a distal tibial fracture extending into the ankle joint, are severe injuries that typically result from high-energy trauma, such as motor vehicle collisions or falls from significant heights. These fractures account for approximately 1-10% of all tibial fractures, with an incidence of 6.5 cases per 100,000 people annually in Western populations [1]. These fractures pose substantial clinical challenges due to their complex anatomical nature and the frequent concomitant soft tissue damage [2]. The primary goal of treatment is the restoration of both anatomical alignment and joint function. A study by Phen et al. in 2019 indicated that proper treatment can reduce the risk of post-traumatic arthritis by up to 30% [3].

The surgical management of pilon fractures has evolved considerably over the past few decades, with numerous techniques employed to achieve stable fixation while minimizing the risk of complications, particularly soft tissue injury. Among the most utilized surgical interventions are external fixation, delayed plating, and open reduction and internal fixation (ORIF) [4]. The selection of treatment modality is often based on the fracture type, degree of soft tissue injury, and the overall health of the patient. Statistics from Daniels et al.'s study indicates that about 45% of pilon fractures are managed with external fixation, 30% with delayed plating, and 25% with ORIF, with varying outcomes [5].

ORIF is the preferred method for fractures that can be treated with early stable fixation and minimal soft tissue involvement [6]. This technique involves direct surgical exposure of the fracture site and the use of plates and screws to achieve rigid fixation. ORIF typically facilitates quicker stabilization and recovery, but it is associated with a higher risk of complications. According to McKissack et al., complication rates for ORIF can reach 25%, with wound infection and non-union being the most common issues [7]. However, ORIF is associated with superior functional outcomes, with study results showing that 80% of patients return to pre-injury activity levels within one year of surgery, compared to 60% in patients treated with external fixation or delayed plating [8].

In contrast, delayed plating involves the use of temporary external fixation or another stabilization method, followed by the delayed application of internal fixation once soft tissue healing has progressed. This approach is believed to reduce the risk of soft tissue complications by allowing adequate time for the tissues to recover before definitive surgical intervention [5].

External fixation is frequently employed in the initial management of pilon fractures, particularly in cases with extensive soft tissue damage. This method allows for temporary stabilization of the fracture while minimizing the risk of exacerbating soft tissue injury [9]. However, external fixation is associated with several potential complications, including pin-site infections, non-union, and joint stiffness. A study by Simpson et al. reported complication rates of up to 30% in patients treated with external fixation, with pin-site infections occurring in 15-20% of cases [10].

Despite the extensive use of these techniques, there remains limited consensus in the literature regarding the optimal treatment modality for pilon fractures, particularly in terms of long-term functional outcomes. In 2016, a study conducted by Duckworth et al. included a group of patients with pilon fractures. The treatment approach included primary ORIF for most of the patients, while others were treated with primary external fixation followed by delayed ORIF or external fixation with limited internal fixation. The study reported a notable number of wound infections. At the long-term follow-up, most patients showed favorable outcomes in terms of functional recovery, with most expressing satisfaction with their treatment [11].

While some studies suggest that ORIF offers superior functional recovery, others highlight the potential benefits of delayed plating in minimizing soft tissue complications [12]. Consequently, further research is required to provide definitive conclusions on the most effective treatment.

This prospective cohort study aims to fill this gap in the literature by systematically comparing the functional outcomes of pilon fractures treated with external fixation, delayed plating, and ORIF.

## Materials and methods

Study design and setting

This prospective cohort study was conducted at the Sancheti Institute for Orthopaedics in Pune, India, between June 2018 and December 2019. The study was approved by the Institutional Review Board, and written informed consent was obtained from all patients who participated in the study.

Inclusion and Exclusion Criteria

The inclusion and exclusion criteria are presented in Table 1.

**Table 1:** Inclusion and exclusion criteria

Inclusion Criteria	Exclusion Criteria
Patients aged 16 years and older	Pathological fractures or fractures associated with bone diseases
Closed, complex intra-articular distal tibial fractures (Pilon fractures)	Patients younger than 16 years
Fractures classified as AO/OTA Types 43-C1 to 43-C3	Open fractures
	History of congenital anomalies or deformities in the affected limb
	Neuropathic foot or significant vascular compromise in the affected limb
	Previous surgery to the affected tibia

Study groups

Patients were assigned to one of three surgical groups based on the treatment approach selected by the treating surgeon, considering the fracture type and soft tissue condition at presentation:

Group A (initial external fixation with delayed plating): External fixation was initially used to stabilize the fracture, followed by plating once soft tissue swelling subsided and skin condition improved (7-21 days later).

Group B (external fixation with some internal fixation): For patients with significant soft tissue injury, external fixation was combined with limited internal fixation (e.g., screws or Kirschner wires) to provide fracture stability.

Group C (one-stage ORIF with plating): In cases with favorable soft tissue conditions, patients underwent ORIF using pre-contoured plates in a single surgical stage.

Variables and measurements

The primary outcome of the study was the functional assessment of the ankle and hindfoot, which was measured using the American Orthopaedic Foot and Ankle Society (AOFAS) Hindfoot Score. This score evaluates pain, movement, and alignment to provide an overall measure of function. Secondary outcomes included the assessment of functional limitations using the Foot and Ankle Disability Index (FADI), which measures how foot and ankle injuries impact daily activities. Additionally, range of motion (ROM) was measured for both dorsiflexion and plantar flexion using a goniometer.

Preoperative care

Upon arrival at the emergency room, all patients were initially treated with fracture manipulation and the application of a back slab to provide temporary stabilization. For patients presenting with significant soft tissue swelling or poor skin conditions, the limb was elevated, and cooling measures were implemented to reduce swelling. A plaster splint was applied for additional immobilization. Surgery was postponed until the swelling subsided, the wrinkle sign (indicating adequate skin turgor) became positive, and the skin condition improved to ensure that the skin was in optimal condition for surgical intervention. This approach helped minimize the risk of complications, such as wound dehiscence or infection, associated with poor skin conditions during surgery.

Surgical procedures

The choice of surgical procedure was based on the fracture type, soft tissue condition, and the surgeon's preference.

Group A: Patients received external fixation initially to stabilize the fracture. This provided time for soft tissue healing, after which internal fixation with plating was performed.

Group B: External fixation combined with limited internal fixation (e.g., screws or Kirschner wires) was used when full internal fixation was not possible due to soft tissue concerns.

Group C: If the soft tissue condition is allowed, a single-stage ORIF with pre-contoured plates was performed. The approach (anterolateral, anteromedial, or posterior incision) depended on the fracture location and type.

Postoperative care

After surgery, all patients received standard postoperative care, including pain management and prophylactic anticoagulation therapy. Rehabilitation began with gentle ankle and foot mobilization exercises as tolerated. Full weight-bearing was deferred until there was evidence of fracture healing on follow-up X-rays. Follow-up visits were scheduled at six weeks, three months, six months, and 12 months post-surgery to monitor recovery and assess functional outcomes.

Statistical methods

Descriptive statistics were used to summarize the baseline demographic and clinical characteristics of the study population. Continuous variables were presented as medians with interquartile ranges (IQRs), due to the non-normal distribution of the data and the small sample size. Categorical variables were summarized as frequencies and percentages.

Since the sample size was less than 30 in each group, non-parametric statistical methods were employed to compare functional outcomes and complications across the three surgical groups at each time point (six weeks, three months, six months, and 12 months).

To compare functional outcomes across the three groups, the Kruskal-Wallis test was used to score at six weeks, three months, six months, and 12 months. Bonferroni correction was also used to adjust the p-values for multiple comparisons. Statistical significance was set at a p-value of <0.05 for all tests.

## Results

Study population

A total of 34 patients were enrolled in the study, with an average age of 41±12.34 years. The sample consisted of 18 males and 16 females. The patients were divided into three surgical groups based on the type of fixation: Group A (n=6), Group B (n=5), and Group C (n=23).

The gender distribution varied between the groups: Groups A and B had a higher proportion of males (70% in Group A and 80% in Group B), while Group C had nearly equal numbers of male and female patients (52% male, 48% female), as shown in Table 2.

**Table 2:** Baseline characteristics of study participants

Characteristic	Group A (n=6)	Group B (n=5)	Group C (n=23)	SMD (A vs B)	SMD (B vs C)	SMD (A vs C)
Age (mean ± SD)	44.01±7.91	41.63± 12.84	32.32 ± 10.4	0.202	0.896	1.064
Male (n, %)	4 (70%)	4 (80%)	12 (52%)	0.212	0.630	0.612
Female (n, %)	2 (30%)	1 (20%)	11 (48%)	0.212	0.630	0.612
AO/OTA type (n, %)	C1: 2 (33%)	C1: 1 (20%)	C1: 10 (43%)	0.236	0.565	0.251
	C2: 2 (33%)	C2: 2 (40%)	C2: 9 (39%)	0.236	0.043	0.191
	C3: 2 (33%)	C3: 2 (40%)	C3: 4 (17%)	0.236	0.565	0.614
Mode of Injury (n, %)						
Road Traffic Accidents (RTAs	4 (66.7%)	2 (40%)	8 (34.8%)	0.649	0.147	0.427
Falls from Height	2 (33.3%)	3 (60%)	7 (47.8%)	0.649	0.630	0.251
Twisting Injuries	0 (%)	0 (0%)	4 (17.4%)	0.000	0.703	0.661

At six weeks and three months, Group C shows significantly better dorsiflexion compared to Group A and Group B. After the Bonferroni adjustment, only the six-week comparison between Group C and the other groups remains significant, as shown in Table 3.

**Table 3:** Range of motion (dorsiflexion)

Timepoint	Group A (n=6)	Group B (n=5)	Group C (n=23)	Unadjusted P-value	Adjusted p-value (Bonferroni)
Median (IQR)					
6 weeks	4.5 (4,5)	5 (4,6)	8 (7,9)	0.012	0.048
3 months	6 (5,8)	6 (5,7)	8.5 (8,9)	0.035	0.140
6 months	10 (9,11)	7 (6,8)	11.5 (11,12)	0.083	0.332
12 months	12 (11,13)	8 (7,9)	13 (12,14)	0.113	0.452

Group C shows significantly better plantar flexion compared to Groups A and B at six weeks and three months. After adjustment, the differences remain significant at six weeks but become non-significant at three months, six months, and 12 months.

This indicates that while ORIF with plating offers an early advantage in plantar flexion, all three surgical methods yield similar results in the long term. This is presented in Table 4.

**Table 4:** Range of motion (plantar flexion)

Timepoint	Group A (n=6)	Group B (n=5)	Group C (n=23)	Unadjusted p-value	Adjusted p-value (Bonferroni)
Median (IQR)					
6 weeks	12 (11,14)	13 (12,14)	17 (16,18)	0.02	0.08
3 months	17 (16,18)	18 (17,19)	21 (20,220	0.029	0.116
6 months	25 (24,26)	23 (22,24)	28 (27,29)	0.094	0.376
12 months	30 (29,32)	29 (28,30)	34 (33,35)	0.145	0.58

Group C shows significantly better FADI scores at six weeks, three months, and six months compared to the other groups. After adjustment, these differences remain significant at six weeks but are no longer significant at three months, six months, and 12 months. This is presented in Table 5.

**Table 5:** Foot and Ankle Disability Index (FADI)

Timepoint	Group A (n=6)	Group B (n=5)	Group C (n=23)	Unadjusted P-value	Adjusted p-value (Bonferroni)
Median (IQR)					
6 weeks	51 (47,54)	43.67 (42,45)	65.87 (62,68)	0.043	0.172
3 months	62.5 (60,65)	54.82 (52,58)	71 (69,73)	0.036	0.144
6 months	76.33 (73,79)	63.66 (61,66)	78.04 (75,80)	0.041	0.164
12 months	86.85 (83,89)	77.88 (75,80)	86.14 (83,89)	0.08	0.32

Group C shows significantly higher AOFAS scores at six weeks, three months, and six months. After Bonferroni adjustment, these differences remain significant at six weeks and six months, but not at three months or 12 months. This is presented in Table 6.

**Table 6:** American Orthopaedic Foot and Ankle Society Hindfoot Score (AOFAS)

Timepoint	Group A (n=6)	Group B (n=5)	Group C (n=23)	Unadjusted p-value	Adjusted p-value (Bonferroni)
Median (IQR)					
6 weeks	54.43 (52,58)	48.67 (47,50)	71.87 (68,74)	0.02	0.08
3 months	65.32 (63,68)	53.76 (51,56)	75 (73,77)	0.032	0.128
6 months	75.6 (73,78)	55.6 (53,58)	78.04 (75,80)	0.02	0.08
12 months	85 (83,88)	76.2 (73,79)	87.04 (85,89)	0.168	0.674

The most common complication observed was delayed wound healing, occurring in eight patients (four in Group A and four in Group C). A pin tract infection was noted in one patient in Group B. Prolonged swelling was recorded in six patients, all in Group C. The majority of patients (19 out of 34) experienced no complications. This indicates that, while each surgical modality has its specific risks, the overall incidence of complications is relatively similar across all groups. This is presented in Table 7.

**Table 7:** Complications

Complication	Group A	Group B	Group C	Total (N=34)
Delayed wound healing n(%)	4 (66.7%)	0 (0%)	4 (17.4%)	8 (23.5%)
Pin tract infection	0 (0%)	1 (20%)	0 (0%)	1 (2.9%)
Prolonged swelling	0 (0%)	0 (0%)	6 (26.1%)	6 (17.6%)
No complication	2 (33.3%)	4 (80%	13 (56.5%)	19 (55.9%)

## Discussion

Our findings reveal that Group C (ORIF with plating) had significantly better early functional outcomes in terms of both dorsiflexion and plantarflexion, as well as superior FADI scores at six weeks and three months compared to Group A and Group B. However, by six and 12 months, the differences across the groups diminished, suggesting similar long-term functional outcomes. We included 34 patients, with 52.9% being male and 47.1% female. The numbers showed no major differences in age or gender among groups.

At six weeks and three months, Group C demonstrated statistically significant improvements in range of motion (dorsiflexion and plantarflexion) and AOFAS scores, compared to the other two groups, with p-values of 0.02 and 0.036, respectively. These results are consistent with a study conducted by Rubio-Suarez et al., highlighting the importance of early fixation and stabilization in the management of pilon fractures. External fixation with delayed plating or limited internal fixation, as used in Group A and Group B, likely delays the restoration of function due to prolonged healing times, particularly in patients with significant soft tissue injury [13]. The initial use of external fixation, while providing temporary stability, may have led to prolonged immobilization and thus delayed recovery of joint function.

The delayed wound healing and prolonged swelling complications observed were most notable in Group A, where 66.7% of patients experienced delayed wound healing, compared to 17.4% in Group C. This supports findings from a study conducted in 2013 by Xu et al., which suggested that, while staged procedures are beneficial in managing soft tissue conditions, they may also be associated with higher rates of complications, including wound dehiscence and delayed healing [14].

In terms of complications, delayed wound healing was the most common issue observed, particularly in Group A, where 66.7% of patients experienced delayed healing, compared to 17.4% in Group C (Table 7). Pin tract infections were observed in one patient in Group B, and prolonged swelling was a complication seen only in Group C (26.1%). These results are consistent with the literature on staged procedures, which often carry a higher risk of complications such as delayed healing and infections, especially in patients with significant soft tissue damage [14]. In this study, despite the early functional benefits in Group C, there were still risks of prolonged swelling, which reflects the complexity of managing soft tissue injuries in pilon fractures. The results suggest that one-stage ORIF with plating (Group C) offers significant advantages in the early recovery of joint motion and function compared to external fixation methods. However, by six and 12 months, the functional outcomes across all groups appear to be similar, which aligns with previous research indicating that the long-term benefits of early versus delayed fixation tend to converge.

Study limitations

The sample size of 34 patients is relatively small, and further studies with larger cohorts are needed to confirm these findings. As the surgery was done at a single center, the results might not apply to other hospitals, where different methods, tools, and patients could influence the results. The follow-up time of 12 months helped look at short- to mid-term recovery but was not long enough to see long-term problems such as late arthritis or issues with the implant that may come up after one year. Additionally, while this study assessed functional outcomes and ROM, it did not evaluate other potential factors, such as patient-reported pain levels or quality of life, which could provide a more comprehensive understanding of the impact of different surgical treatments. Furthermore, the non-randomized allocation of the patients in treatment groups based on the doctor's clinical led to possible bias. This method, influenced by factors such as swelling and skin condition, may have led to more complex cases getting staged treatment, influencing the results. Different types of fractures, surgical methods of rehabilitation, and patient adherence to physical therapy could have caused differences in the findings. Future research should fix these issues by using larger, multi-center random trials with longer follow-up times and better tracking of rehab efforts.

## Conclusions

This study evaluated the outcomes of different surgical approaches for pilon fractures, specifically comparing primary ORIF with plating, initial external fixation with delayed plating, and external fixation with some internal fixation. Our results indicate that Group C (ORIF) demonstrated significant short-term advantages at six weeks and three months for range of motion (dorsiflexion and plantar flexion) and functional outcomes (FADI and AOFAS scores). These early benefits are likely due to the ability of ORIF to restore joint stability and facilitate an earlier start to rehabilitation. However, by six months and 12 months, the differences in outcomes between the groups became non-significant, suggesting that the long-term benefits of ORIF were not markedly superior to the other treatments. Group A (external fixation) and Group B (two-stage procedure) showed similar long-term functional recovery, indicating that all three approaches can provide satisfactory outcomes in the long term. The results highlight the importance of choosing a surgical approach based on fracture characteristics, soft tissue condition, and patient-specific factors, rather than solely focusing on the short-term benefits of ORIF. Ultimately, while ORIF offers a short-term advantage, all surgical approaches provide comparable long-term functional outcomes, and decisions regarding treatment should be individualized to optimize recovery and minimize complications.
